# Application of Next-Generation Sequencing in Infections After Allogeneic Haematopoietic Stem Cell Transplantation: A Retrospective Study

**DOI:** 10.3389/fcimb.2022.888398

**Published:** 2022-06-14

**Authors:** Xiaoying Zhang, Yun Li, Jin Yin, Bixin Xi, Na Wang, Yicheng Zhang

**Affiliations:** ^1^Department of Hematology, Tongji Hospital, Tongji Medical College, Huazhong University of Science and Technology, Wuhan, China; ^2^Department of Pediatrics, Tongji Hospital, Tongji Medical College, Huazhong University of Science and Technology, Wuhan, China; ^3^Institute of Organ Transplantation, Tongji Hospital, Tongji Medical College, Huazhong University of Science and Technology, Wuhan, China

**Keywords:** next-generation sequencing, allogeneic haematopoietic stem cell transplantation, diagnosis, infection, immunosuppression

## Abstract

This retrospective study aimed to determine the characteristics of infection and diagnostic efficacy of next-generation sequencing (NGS) in patients with fever after allogeneic hematopoietic stem cell transplantation (allo-HSCT). A total of 71 patients with fever after HSCT were enrolled in this study. Compared with conventional microbiological test (CMT), we found that the sensitivity of NGS versus CMT in peripheral blood samples was 91.2% vs. 41.2%, and that NGS required significantly less time to identify the pathogens in both monomicrobial infections (*P*=0.0185) and polymicrobial infections (*P*= 0.0027). The diagnostic performance of NGS was not affected by immunosuppressant use. Viruses are the most common pathogens associated with infections. These results indicated that the sensitivity, timeliness, and clinical significance of NGS are superior for the detection of infections. Although NGS has the advantage of identifying a wide range of potential pathogens, the positive rate is related closely to the sample type. Therefore, we recommend that, in the clinical application of NGS to detect pathogens in patients after allo-HSCT, an appropriate sample type and time should be selected and submitted to improve the positive rate and accuracy of NGS. NGS holds promise as a powerful technology for the diagnosis of fever after HSCT.

## Introduction

Allogeneic hematopoietic stem cell transplantation (allo-HSCT) is a curative option for a wide range of disorders such as hematological malignancies and some nonmalignant diseases (Blazar et al., 2020). Infections are one of the most frequent and important causes of mortality and morbidity after allo-HSCT (Styczyński et al., 2020). Allo-HSCT is a complicated and multifactorial process, in which the standard risks are associated mainly with neutropenia, mucositis, and catheter use. In addition, myeloablative conditioning regimens, reconstitution of the immune system, use of immunosuppressive drugs, and graft-versus-host disease (GVHD) are independent risk factors for infections (Kao and Holtan, 2019). Infectious pathogens have also varied among the studies that have used different testing methods. Conventional microbiological tests remain the main methods used to identify pathogens in the clinic, such as smear microscopic examination and culture; however, these are relatively insensitive and are used mainly to detect bacteria, fungi, and parasites (Goldberg et al., 2015; Grumaz et al., 2016; Gu et al., 2019). Recently, nucleic acid amplification techniques, such as real-time quantitative polymerase chain reaction (RQ-PCR) and multiplex PCR, have been used widely for the diagnosis of infectious diseases, especially viral infections (Watzinger et al., 2006; Schlaberg et al., 2017b). Multiplex PCR has been recognized as the “gold standard” to identify viruses in central nervous system(CNS) infections (Schmidt-Hieber et al., 2016). Nevertheless, there are still some limitations to the use of RQ-PCR, such as the genetic diversity of some pathogens and the need for knowledge of the target pathogen (Schlaberg et al., 2017b). Therefore, there is an urgent need to develop novel diagnostic methods to detect potential pathogens in undetermined infections successfully.

Next-generation sequencing (NGS) has showed a constant improvement in recent decades with its continuous improvements and use in clinical settings, providing a powerful tool for success in medical practice (Goldberg et al., 2015; Wilson et al., 2019; Tang et al., 2021). NGS is capable of detecting multiple pathogens on the same day at the same time rapidly (Barreda-García et al., 2018), includingthe identification of nonculturable microbes (Íñigo et al., 2016). Recent studies have identified pathogens using NGS in the diagnosis of several diseases, including diseases of the respiratory tract (Huang et al., 2020; Li et al., 2020), urinary tract (Gasiorek et al., 2019), central nervous system (CNS) (Schmidt-Hieber et al., 2016; Liu et al., 2021), bloodstream (Grumaz et al., 2016; Eichenberger et al., 2021; Nie et al., 2022), and periprosthetic joint infections (Tarabichi et al., 2018). However, there have been relatively few studies on the use of NGS for infections after allo-HSCT. Clinical experience with the application of NGS is relatively limited. Due to the severity and uniqueness of infections in patients after allo-HSCT, the rapid and accurate diagnosis and assessment are important for rational treatment and prognosis evaluation (Sahin et al., 2016). This study aimed to compare the efficacy of NGS with that of conventional microbiological test (CMT) and to determine whether NGS technology can meet this need by evaluating its ability to detect pathogens in febrile patients after allo-HSCT. At the same time, the infection status of the patients after HSCT was evaluated.

## Material and Method

### Patients and Study Design

We retrospectively analyzed the basic situation, pathogenic infections, clinical treatment, and prognosis of 71 patients who underwent allo-HSCT at the Hematopoietic Stem Cell Transplantation Center of Tongji Hospital, affiliated with the Huazhong University of Science and Technology between March 2019 and October 2020. These patients developed fever with or without other symptoms after transfusion and who underwent CMT and NGS tests. Different specimen types were collected for detection, according to the type of suspected infection. The clinicians prescribed CMT according to their clinical judgment of necessity. The CMT included smear microscopy, culture, RQ-PCR, T-SPOT TB test, serological tests, and the detection of antigens (**Supplementary Methods**). The samples were collected and transported to Huada Laboratories (Shenzhen, China) for NGS. The treatment choice was individualized for each patient.

### Infection Prophylaxis and Virus Monitoring

All the patients were treated in a transplantation cabin. Antimicrobial prophylaxis was used routinely: voriconazole 0.4 g once every 12 h, acyclovir 400 mg once every 12 h, and trimethoprim-sulfamethoxazole 960 mg twice a day for two days each week. When an infection was suspected, the attending physician adjusted the antimicrobial protocols according to the patients’ conditions and the institutional guidelines. Ciclosporin A (CsA) and tacrolimus (FK-506) were generally used to treat GVHD. RQ-PCR was used to measure the DNA of the Epstein–Barr virus (EBV) and cytomegalovirus (CMV) in the peripheral blood (PB) weekly for the first three months, then once every two weeks from the 4th to the 9th month and then once per month from the 10th to the 12th month after transplantation.

### Diagnosis of Infection

Fever was defined as an axillary temperature ≥37.3°C. The diagnosis was based on clinical symptoms, laboratory tests, radiographic, microbiologic, histopathologic findings, and treatment outcome information. The final diagnosis of infections was performed by two independent experienced clinicians according to published consensus criteria (Ascioglu et al., 2002; De Pauw et al., 2008; Haidar and Singh, 2022). The appropriate clinical specimens were collected for testing (i.e., blood samples, sputum, urine, nasopharyngeal swabs, puncture fluids, tissue samples, aspirates, and bronchoalveolar lavage fluid) according to the type of suspected infection. The site of infection included mainly the bloodstream, respiratory tract, CNS, and skin. The day of infection onset was defined as the day on which the diagnostic test was performed. Multiple positive results for different organisms on the same day were considered as separate events, and one microorganism in two non-adjacent organs was counted as two infectious events. In addition, if the detected microorganism was a possible contaminant (for example, Candida in a fecal culture or coagulase-negative Staphylococcus species in a blood culture) and was isolated in only one culture, it was excluded from the analysis.

### NGS Procedure

Qualified sample from patient was collected and stored according to standard procedures. After DNA extraction, the DNA libraries were constructed and sequenced by MGISEQ-2000 platform. Next, High-quality sequencing data were generated by removing low-quality and short (length <35 bp) reads, followed by computational substraction of human host sequences mapped to the human reference genome (hg19) using Burrows-Wheeler Alignment. The remaining data by removal of low-complexity reads were classified by simultaneously aligning to Pathogens metagenomics Database (PMDB), which were downloaded from the NCBI (ftp://ftp.ncbi.nlm.nih.gov/genomes/) (Schoch et al., 2020), consisting of bacteria, fungi, viruses and parasites.

To eliminate false positive, periodic environmental assessments was conducted, and standard operation Procedures (SOP) was established to monitor contamination for periodic disinfection of reagents, instruments, and laboratory surfaces. Possible contaminants in non-template references or positive controls were continuously tracked, and conservative criteria was used to minimize false positive results. As for the cut-off value of NGS for pathogen detection, the criteria were shown as below: 1) Bacteria (mycobacteria excluded), viruses, and parasites: NGS identified a microbe (species level) whose coverage rate scored 10-fold greater than that of any other microbes according to Langelier’s study. 2) Fungi: NGS identified a microbe (species level) whose coverage rate scored 5-fold higher than that of any other fungus because of its low biomass in DNA extraction. 3) Mycobacteria: Mycobacterium tuberculosis (MTB) was considered positive when at least 1 read was mapped to either the species or genus level due to the difficulty of DNA extraction and low possibility for contamination.

### Statistical Analysis

All the clinical and laboratory data were collected during the onset of the infection. The sensitivity, specificity, positive predictive value (PPVs), and negative predictive value (NPVs) were calculated according to the definitions. The Chi-square or Fisher’s exact tests were used for categorical variable comparisons, as appropriate. The kappa (κ) statistic was used to assess the test concordance. The student’s t-test or Kruskal–Wallis test was used for the continuous variables, as appropriate. Statistical analyses were conducted using SPSS version 26.0 (IBM Corp., Armonk, NY, USA), and figures were rendered using GraphPad Software (version 8.02; Mariakerke, Belgium) and R Software (version 3.6.3). The statistical significance set at *P* <0.05 (two-tailed) was considered to be statistically significant.

## Results

### Patients’ Characteristics and Infection Among All Patients

Thirty-seven patients were male and 34 were female, and the patients had a median age of 23 years (range, 14–33). The primary diseases included acute myeloid leukemia (AML, n = 29), acute lymphoblastic leukemia (ALL, n = 15), myelodysplastic syndrome (MDS, n = 3), aplastic anemia (AA, n = 22), and T lymphoblastic lymphoma (T-LBL, n = 2). Thirteen patients were HLA-matched, and 58 underwent HLA-mismatched donor transplantation. All the patients received myeloablative conditioning regimens. At the onset of the symptoms, 23 patients (32.2%) had agranulocytosis and 58 (81.7%) received immunosuppressive therapy. The patient samples comprised mainly peripheral blood (57.7%). The median values of CRP, PCT, and IL-6 in all of the patients were 70.4 (IQR, 20.7–148.6), 0.445 (IQR, 0.263–1.378), and 50.22 (IQR, 7.20–124.00), respectively. The baseline clinical and biochemical data of the 71 patients are described in **Table 1**.

**Table 1:** Patient characteristics.

	All patients (n = 71)
Age, years (median, IQR)	23 (14-33)
Female	37 (52.1%)
Protopathy
AA	22 (30.1%)
ALL	15 (21.1%)
AML	29 (40.8%)
MDS	3 (4.2%)
T-LBL	2 (2.8%)
Transplantation way
Haplo	58 (81.7%)
MSD	6 (8.5%)
MUD	7 (9.9%)
N engraftment, days (median, IQR)	13 (11-15)
PLT engraftment, days(median, IQR)^*^	13 (12-16)
Main Symptoms
fever	50 (70.4%)
diarrhea	8 (11.3%)
cough	3 (4.2%)
rash	3 (4.2%)
blurred version	1 (1.4%)
headaches	3 (4.2%)
ascites	1 (1.4%)
tic	1 (1.4%)
Sample collection time
Peri-planting period	12 (16.9%)
<100D	25 (35.2%)
>100D	34 (47.9%)
Agranulocytic	23 (32.4%)
Immunosuppressive drugs	58 (81.7%)

PLT engraftment, days (median, IQR)*: excluded 3 cases without PLT engraftment.

AA, aplastic anemia; ALL, Acute lymphoblastic leukemia; AML, Acute myeloid leukemia; MDS, Myelodysplastic syndromes; T-LBL, T lymphoblastic lymphoma; Haplo, Haploidentical stem cell transplantation; MSD, Matched sibling donor; MUD, Matched unrelated donor.

**Figure 1:**
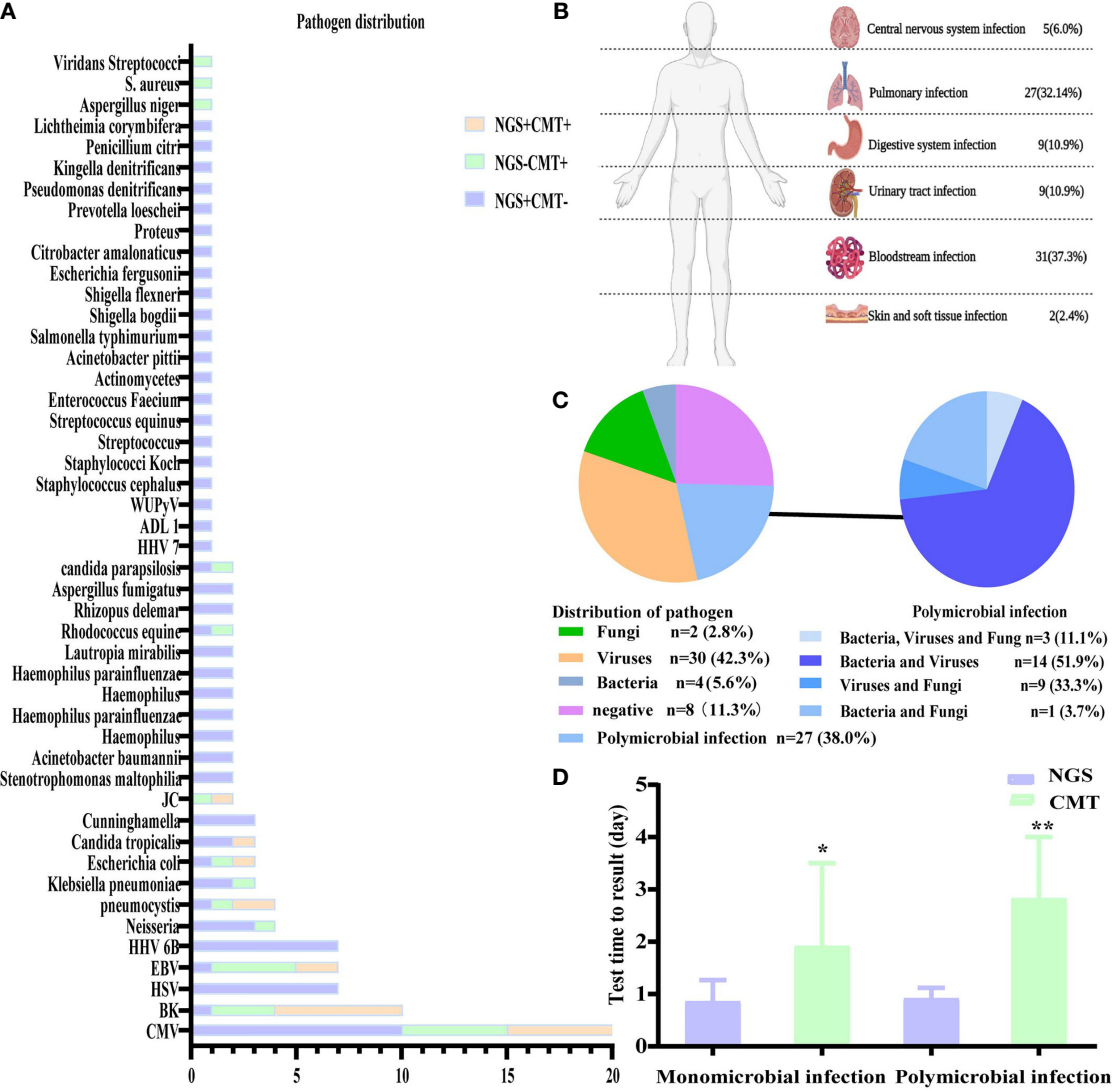
Distribution of pathogens identified in patients with fever after allo-HSCT using CMT versus NGS. **(A)** The figure showed the number of subjects in whom each causative microbe was detected. Orange bars indicate microbes detected by CMT and also predicted as pathogens by NGS (CMT+NGS+). Purple bars indicate microbes detected by NGS only (CMT−NGS+). Green bars indicate the number of cases with microbes detected only by CMT (CMT+NGS−); **(B)** distribution of types of infection was shown from patients with clinical diagnosis; **(C)** distribution of pathogens was shown from patients. Polymicrobial infection accounted for 38.0% among all the subjects and different kinds of polymicrobial infection were also shown in the right; **(D)** the diagnostic time required for NGS and CMT were compared in subjects with monomicrobial infection or polymicrobial infection. *P<0.05; **P<0.001 by Wilcoxon rank-sum test.

According to the consensus criteria, the diagnosis was confirmed in 54 patients. Only one patient’s NGS and CMT results were negative, but based on the subsequent pathological biopsy results, the patient was diagnosed with an invasive fungal infection. Most of the patients’ infections were viral, followed by polymicrobial infections. The infections were present mainly in the bloodstream, followed by pulmonary, digestive, and urinary tract infections. Five patients were diagnosed with central infections (diagnosed using intracranial biopsy or cerebrospinal fluid analysis), and two patients were diagnosed with skin and soft tissue infections (**Figure 1B**).

### Diagnostic Performance of NGS and CMT

The comparison of the sensitivity, specificity, PPVs, and NPVs of the peripheral blood samples by NGS and the CMT method for all 41 patients is shown in **Table 2**. The diagnostic value of NGS was significantly higher than that of CMT (P < 0.001). The results of NGS and CMT were concordant in 15 of the 41 (36.6%) patients. Our results showed that the sensitivity of NGS and CMT were respectively 91.2% and 41.2% for pathogen identification in the PB samples of 41 patients.

**Table 2:** Comparison of positive results among next-generation sequencing and conventional microbiological tests.

		Positive	Negative	*P <0.001*
NGS	Positive	31	5	
	Negative	3	2	
CMT	Positive	14	2	
	Negative	20	5	
	Sensitivity%	Specificity%	PPV	*NPV*
NGS	91.2	28.6	0.861	0.4
CMT	41.2	71.4	0.875	0.8

Positive, patients with a positive clinical diagnosis; NGS, next-generation sequencing; CMT, conventional microbiological tests.

In the present study, the pathogens of NGS and CMT detected in the patients are shown in **Figure 1A**. Viruses, especially CMV, which was the highest percent, accounted for the majority of the pathogens, and NGS was better for the detection of rare bacteria and fungi. According to the NGS results, viruses (n=30, 42.3%) were the most common pathogens identified, followed by polymicrobial (**Figure 1C**). The top two causative pathogens identified were CMV (n=20), BK (n=10). We further analyzed the test time required to determine the pathogenic diagnosis. For monomicrobial infections and polymicrobial infections, the detection cycles required for NGS and CMT were significantly different. CMT required significantly more time to identify the pathogens than NGS (*P* = 0.0185, *P*=0.0027) (**Figure 1D**). Meanwhile, we analyzed the detection performance of NGS and CMT in monomicrobial and polymicrobial infections. The sensitivity of NGS and CMT were 81.58% vs 57.89% (*P* = 0.0445) for monomicrobial infections, respectively, and 68.75% vs 37.50% (*P*=0.1556) for polymicrobial infections, respectively. This indicated that NGS was more sensitive than CMT for monomicrobial infections and a suggestive, but not significant benefit in polymicrobial infections.

### Distribution of the Infections in the Different Periods After Transplantation

**Figure 2:**
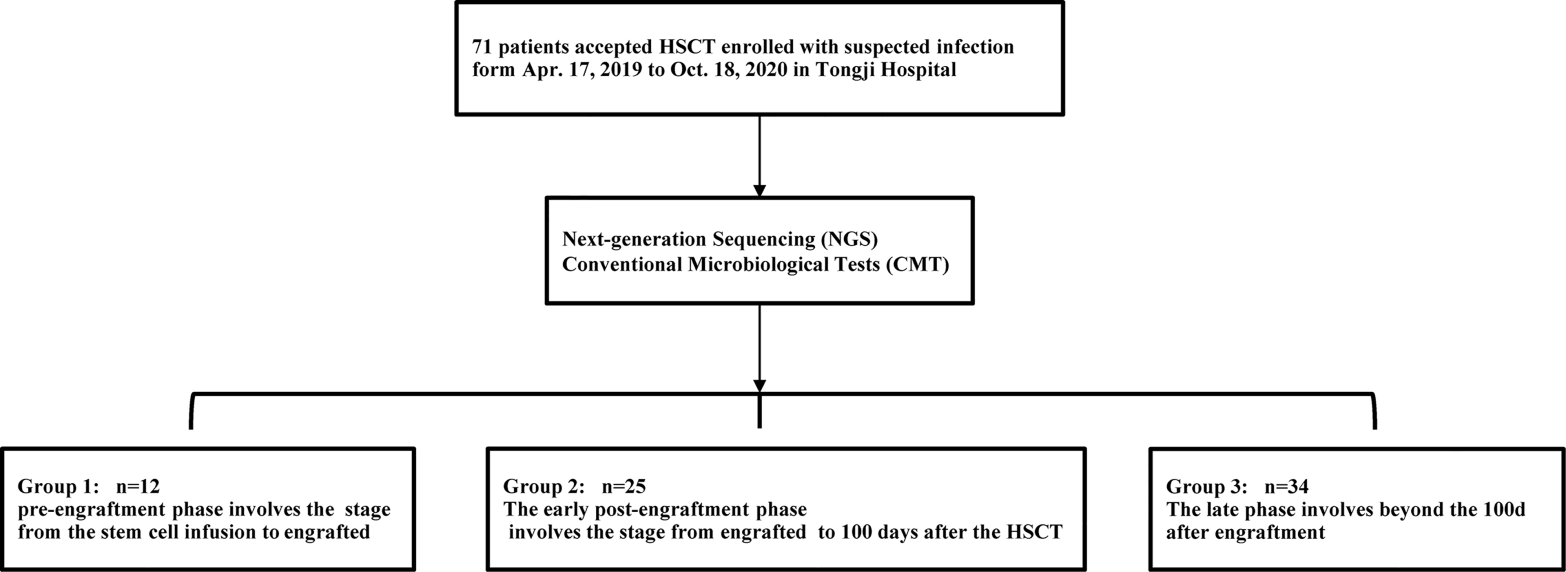
Trial process. Between Apr.17, 2019 to Oct.18, 2020, 71 patients who developed fever with/without other symptoms after the transfusion and underwent CMT and NGS tests were screened for eligibility in this study. In terms of the time of clinical symptoms appeared, the patients were divided into three groups.

**Figure 3:**
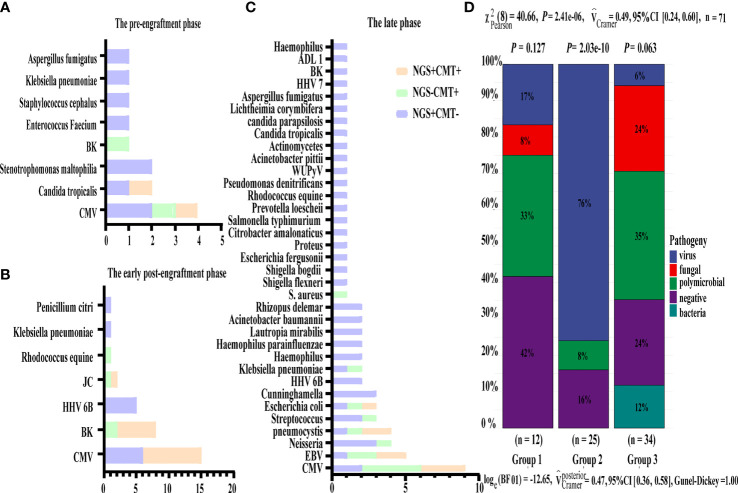
Distribution of pathogens identified in three group using CMT versus NGS. **(A–C)** Respectively showed the number of subjects in whom each causative microbe was detected in three group. Orange bars indicate microbes detected by CMT and also predicted as pathogens by NGS (CMT+NGS+). Purple bars indicate microbes detected by NGS only (CMT−NGS+). Green bars indicate the number of cases with microbes detected only by CMT (CMT+NGS−). **(D)** Distribution of pathogens was shown from patients in three group. Virus infection accounted for 76% among group 2 and there were significant differences in pathogen species.

Based on the occurrence of symptoms in the different periods after transplantation, the 71 patients were divided into three groups (**Figure 2**). The male-to-female ratio, primary disease, mode of transplantation, and complications of GVHD in the three groups are shown in **Supplementary Table 1**. Among them, seven and nine patients in Groups 2 and 3, respectively, were complicated with GVHD at the time of infection. The positive pathogens are shown in **Figures 3A–C** with CMV being the most common infection. Patients with Aspergillus fumigatus in Group 1 had a history of fungal infection before transplantation. In Group 2, there was a high detection rate of cystitis with the detection of BK and JC viruses in the urine. Group 3 had more complex pathogenic pathogens, fungal species, and rare bacterial species. The detection results for fever and other infectious symptoms in Group 1 were mainly negative. The main reason for this is that the disease was diagnosed as an ALG-related serum sickness and implantation syndrome, and the results of NGS and CMT were used as exclusion tests. Second, it may have been an immune disorder in patients with peri-implantation and a low pathogen detection rate. In Group 2, the pathogens that were detected were mainly viruses (*P* = 0.0203), especially CMV; an HHV6 infection also needed special attention. In Group 3, fungal, viral, mixed, and rare pathogens accounted for a relatively high proportion of the pathogens, which may have been related to late post-transplantation patients with GVHD and other transplantation-related complications, patients taking immunosuppressants, and other reasons (**Figure 3**).

### No Significant Influence of Immunosuppression on the Diagnostic Accuracy of NGS and CMT

**Figure 4:**
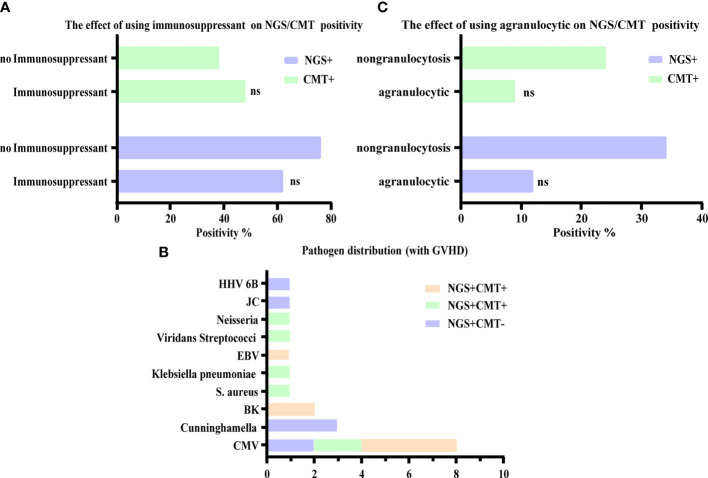
Influence of other factors on the diagnostic accuracy of NGS and CMT. **(A, C)** Respectively shown that the positive microbiological detection rate of NGS and CMT was no significant correlation with immunosuppression and agranulocytic. **(B)** The number of detected microbes in patients with GVHD were presented, of which mainly were CMV. ns, non-significant statistical difference.

To determine whether the diagnostic performance of NGS was affected by immunosuppression, we evaluated the relationship between immunosuppression and the positive rate of CMT and NGS. Our results showed that there was no significant difference in the positivity rate between NGS and CMT, regardless of the use of immunosuppressants. That is, the positivity rate was not affected by the use of immunosuppressants (**Figure 4A**). In addition to the routine use of immunosuppressants according to the anti-GVHD regimen, patients after HSCT will need to have their dosage and usage of immunosuppressive mediations adjusted individually when GVHD occurs. Therefore, we investigated the distribution of pathogens in GVHD patients. CMV infection was also the most common, of which six cases were intestinal GVHD coinfected with gastrointestinal CMV infections, and NGS had excellent performance in diagnosing HHV 6 B and Cunninghamella (**Figure 4B**). At the same time, we analyzed the positive results of NGS and CMT in patients with agranulocytosis, but also found no significant effect (**Figure 4C**).

### Effect of the Different Specimen Types on the NGS and CMT Results

**Figure 5:**
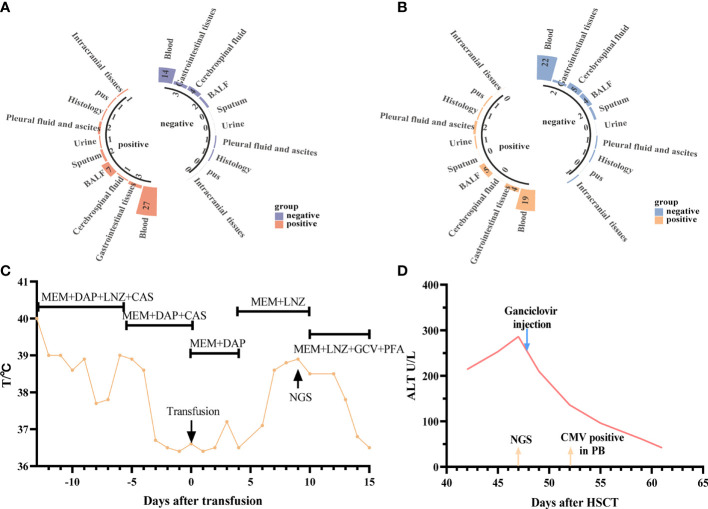
Diagnostic value of NGS and CMT in different specimens. **(A, B)** Respectively shown that the positive microbiological detection rate of NGS and CMT using different specimens **(C)** representative case 1. A 5-year-old male patient with SAA developed a fever after hematopoietic stem cell (HSC) infusion. Meropenem and Daptomycin was given based on previous treatments but the symptom was not improved after 7 days of treatment. The CMT had negative results both blood and urine. Then the anti-infective regimen was adjusted according to NGS results (HHV-6B), which was obtained at +9d. After 7-day’ s therapy the patient’ s symptoms were improved; **(D)** representative case 2. A 22-year-old female patient with SAA developed low fever and persistent ALT elevation, and had no positive results in various laboratory tests. The liver puncture was performed, she was diagnosed with CMV infection (liver) based on the results from NGS at +47d. And then modified as soon as the NGS results were obtained (Ganciclovir). After only 5 days, CMV copy number was detected in peripheral blood. The patients’ symptoms were finally improved.

The distribution of pathogens in different tissues, organs, and systems is different; therefore, the type of samples submitted for examination will be related closely to the test results. Six patients had different NGS and CMT samples and different detection results, which helped obtain a clearer diagnosis and identify the pathogen of potential infection (**Table 3**). The treatment details for the two representative cases are shown in **Figures 5C, D**. We can draw conclusions from these cases because of the distribution of pathogens in the blood, tissue, or cross, and the appropriate timing is also of great significance in improving the positive rate of detection. In addition, we found that, except for the timing of the examination, the selection of samples was crucial for the detection of a positive rate. The detection rates of NGS and CMT in the different inspection specimens are shown in **Figures 5A, B**, respectively. The CMT of peripheral blood samples, especially the positive rate of the blood cultures, was not as high as that of NGS, but for common pathogens in diseased tissues, such as in the gastrointestinal mucosa of patients with diarrhea and sputum/bronchoalveolar lavage fluid samples of patients with cough, the positive rates of NGS and CMT are similar.

**Table 3:** Details of patients with different types of samples.

ID	Symptoms	Clinical diagnosis	NGS (pathogen, the sequence)	Specimen types	CMT Restult	Specimen types
P1	fevers, rash and cutaneous ulcer	cGVHD(Skin), skin soft tissue infection, EBV syndrome	HSV1 156;EBV 26; CMV 10	Peripheral blood	S. aureus, Klebsiella pneumoniae	secretions
P2	fever and cough	Pulmonary infection, human herpesvirus 6B infection	HHV-6B 517;	Peripheral blood	Klebsiella pneumoniae	sputum
P3	fever with cough, sputum and diarrhea	aGVHD(Gastrointestinal),CMV gastroenteritis, pulmonary infection.	HSV1 3738; HHV-6B 20; HHVB 7type 568; CMV 7; Torque teno virus 25;	Intestinal tissue	Viridans Streptococci, Neisseria	Fiber bronchoscope rinse solution
P4	Low fever and abdominal pain	CMV gastroenteritis, CMV hyperemia, cystitis	CMV 137; BK 10; EBV 3	Peripheral blood	BKV;CMV	Urine;Gastric mucosa
P5	Intermittent low fever	Pneumocystis carinii pneumonia	EBV 6	pleural effusion	Pneumocystis carinii	Fiber bronchoscope rinse solution
P6	fever with cough, sputum	pulmonary infection	Neisseria 3155; Haemophilus parainfluenzae 2579; Haemophilus 21; Prevotella loescheii 680; Rhodococcus equine 278; Pseudomonas denitrificans 10; Kingella denitrificans 114; WUPyV 9009; CMV 1485; HSV1 1326; HHV 7 73; EBV 53680; Rhodococcus equine 278; Pseudomonas denitrificans 10; Kingella denitrificans 114; WUPyV 9009; CMV 1485; HSV1 1326; HHV 7 73; EBV 53	sputum	negative	Peripheral blood

GVHD, graft versus host disease.

## Discussion

Fever after allo-HSCT is complicated. Infection after transplantation is one of the leading causes of fever and other symptoms, and is the most common and important cause of death. Early diagnosis and timely treatment are considered to be the most critical factors in determining the outcomes. Conventional microbiological test (CMT) are obtained when clinicians make a series of differential diagnoses according to the clinical manifestations of cases; however, usually, a test can only correspond to one pathogen, the coverage rate is low, and difficult to cultivate (Duan et al., 2021). Different pathogens have different culture times (Miao et al., 2018). Recently, NGS has become a sensitive technology for the detection of pathogens in human biopsy samples and body fluids, including blood, urine, cerebrospinal fluid, and bronchoalveolar lavage fluids (Huang et al., 2021), demonstrating the potential of NGS to accelerate and improve the diagnosis and management of diseases.

Pendleton et al.demonstrated that, in principle, real-time metagenomics methods using currently available tools can identify pathogens faster than traditional culture-based techniques and have the potential to identify pathogens that cannot grow in cultures (Pendleton et al., 2017). A study on the application of NGS in 108 patients with suspected infections treated with immunosuppressive corticosteroids showed that the sensitivity of NGS was 80.6%, which also played an important role in optimizing the antibiotic treatment of CMT-negative patients (Wang et al., 2020). Moreover, NGS results were not affected by immunosuppression. The combination of NGS and conventional methods increased the CSF detection rate in patients with tuberculous meningitis to 95.65%. They believed that NGS was an alternative method for detecting the presence of mycobacterial DNA in CSF samples of TBM patients and may be used as a first-line CSF test (Wang et al., 2019).

However, there have been few studies on the detection of NGS infections after HSCT. Therefore, we conducted a retrospective study in patients from our center who developed fever after HSCT between March 2019 and October 2020, and 71 patients were tested using NGS. Most of the patients’ infections were viral, followed by polymicrobial infections. For both monomicrobial and polymicrobial infections, the detection time of NGS is significantly shorter than that of CMT, and NGS showed a higher sensitivity, especially for monomicrobial infections. Probably, just because of the small sample size, this has not yet become noticeable in the polymicrobial infections Accelerated pathogen identification is expected to improve customized antimicrobial therapy, avoid increased antibiotic resistance, improve patient prognosis, and promote antimicrobial management by minimizing the need for extensive empirical antimicrobial coverage (Dumford and Skalweit, 2016; Cazzola et al., 2017; Pendleton et al., 2017; Robilotti et al., 2017). CMT may not be comprehensive for some rare pathogens (Grumaz et al., 2020), which may lead to a detection delay, whereas NGS can sequence the whole DNA/RNA of the sample without any primers or probes. It has a commitment to identifying the most pathogens. Among the 41 patients with peripheral blood samples, 34 were diagnosed clinically with an infection. The sensitivity of NGS versus CMT in peripheral blood after transplantation was 91.2% and 41.2%, respectively, which was higher than that of CMT. However, the specificity of NGS is not as specific as that of CMT, which may be attributed to the differences in specimen types and cut-off values. This suggests that caution should be exercised when interpreting NGS results alone, for clinical use. False-positive results may be due to the DNA contamination of background pathogens included in library preparation, low-quality readings from samples, misannotated species, or contaminants from database entries (Miller et al., 2019; Zhang et al., 2019; Zhang et al., 2020). In addition to establishing standardized operating rules, it is necessary to interpret the results further and reasonably.

In the groups studied, although the patients who were post-HSCT were most likely to develop viral infections, the distribution of pathogens was different in different periods. We found that the main causes of fever in the peri-implantation period were non-infectious diseases, such as ALG serum disease and implantation syndrome. After engraftment, the patients who had early infections had mainly viral infections, including CMV infections and cystitis caused by BK and JC. In the late stage after transplantation, the causes of fever and infection varied, and viral and mixed infections occurred first and second, respectively. Regardless of the period, the primary pathogenic viral infection was CMV, which showed that more attention should be paid to the detection and prevention of CMV in clinical practice. More often, NGS plays a major role in ruling out infection in the early post-transplantation period, while in the late post-transplantation period, the role of NGS is more accessible to identify the causative pathogen, especially after 100 days of transplantation. In addition, our results showed that the use of immunosuppressants or agranulocytosis does not affect the NGS detection results. Patients with GVHD may have compromised immune systems. NGS is also an excellent method for detecting pathogens in patients during this period. Intestinal GVHD patients co-infected with gastrointestinal CMV infections were also more common in our study. For patients with normal immune function and stable vital signs, CMT may be used to detect and retain samples simultaneously; if no positive result is found or if the patient’s symptoms have not been alleviated within three days, NGS should be submitted immediately. However, patients with unstable signs or those in an immunosuppressive state should undergo simultaneous CMT and NGS tests. Standardizing the examination process using these diagnostic methods is important to identify pathogens and carry out effective targeted treatment (**Supplementary Figure 1**).

NGS can be used to detect a wide range of pathogens. There is no limitation to the use of one CMT to detect a single pathogen. This also reduces the mixed-packed testing methods of multiple laboratory methods. Packaged laboratory testing methods often require patients to undergo multiple tests, regardless of the cost or number of tests, which will not reduce the burden on patients and may even increase the burden on the patients (Miao et al., 2018). It is more sensitive to rare bacteria and fungi, and its detection period is short. However, because of false positives (Schlaberg et al., 2017a; Blauwkamp et al., 2019), the use of NSG requires strict standardization of detection techniques and an accurate interpretation of the results by clinicians to avoid overtreatment while treating the patient’s diseases (Fan et al., 2018).

This study had several limitations. First, due to its high cost of detection, NGS is usually performed only once per patient, which limits its widespread use and repeated testing. In addition, since this was a retrospective study, the time and tissue samples used for NGS were not precisely the same as those used for CMT, and the time of NGS specimen collection may have been later than that of CMT. As the collection time of NGS may have been different from the time that had the highest number of pathogens in the samples, repeated NGS tests may increase the sensitivity. Second, the sample size in our study was relatively small. To verify our conclusions, we have conducted a prospective study with a larger sample size. Finally, NGS could not perform a drug susceptibility test and, therefore, could not be substituted for conventional testing.

In summary, NGS has the potential to detect pathogens in patients undergoing febrile allogeneic hematopoietic stem cell transplantation. NGS is expected to become a valuable tool in the first-line diagnosis of infection and to provide valuable information for optimizing antibiotic treatment in cases.

## Data Availability Statement

The datasets presented in this study can be found in online repositories. The name of the repository and accession number can be found below: NCBI: PRJNA820385.

## Ethics Statement

Ethical review and approval was not required for the study on human participants in accordance with the local legislation and institutional requirements. Written informed consent from the participants’ legal guardian/next of kin was not required to participate in this study in accordance with the national legislation and the institutional requirements.

## Author Contributions

YZ and NW designed and supervised the clinical study, who have contributed equally to this work and share corresponding authorship. XZ and performed statistical analyses and wrote the manuscript. YL collected clinical data. JY and BX were responsible for diagnosis and patient management. YZ and NW revised the manuscript; All authors contributed to the article and approved the submitted version.

## Funding

This study was supported by the National High Technology Research and Development Program of China (2021YFA1101504) and the National Natural Science Foundation of China (Grant No. 81873446, 82070217, 81600120 and 81873427).

## Conflict of Interest

The authors declare that the research was conducted in the absence of any commercial or financial relationships that could be construed as a potential conflict of interest.

## Publisher’s Note

All claims expressed in this article are solely those of the authors and do not necessarily represent those of their affiliated organizations, or those of the publisher, the editors and the reviewers. Any product that may be evaluated in this article, or claim that may be made by its manufacturer, is not guaranteed or endorsed by the publisher.

## References

[B1] Ascioglu S. Rex J. H. De Pauw B. Bennett J. E. Bille J. Crokeart F.. (2002). Defining Opportunistic Invasive Fungal Infections In Immunocompromised Patients With Cancer And Hematopoietic Stem Cell Transplants: An International Consensus. Clin. Infect. Dis. 34, 7–14. doi: 10.1086/323335 11731939

[B2] Barreda-García S. Miranda-Castro R. De-Los-Santos-Álvarez N. Miranda-Ordieres A. J. Lobo-Castañón M. J. (2018). Helicase-Dependent Isothermal Amplification: A Novel Tool In The Development Of Molecular-Based Analytical Systems For Rapid Pathogen Detection. Anal. Bioanal. Chem. 410, 679–693. doi: 10.1007/S00216-017-0620-3 28932883 PMC7079856

[B3] Blauwkamp T. A. Thair S. Rosen M. J. Blair L. Lindner M. S. Vilfan I. D.. (2019). Analytical And Clinical Validation Of A Microbial Cell-Free Dna Sequencing Test For Infectious Disease. Nat. Microbiol. 4, 663–674. doi: 10.1038/S41564-018-0349-6 30742071

[B4] Blazar B. R. Hill G. R. Murphy W. J. (2020). Dissecting The Biology Of Allogeneic Hsct To Enhance The Gvt Effect Whilst Minimizing Gvhd. Nat. Rev. Clin. Oncol. 17, 475–492. doi: 10.1038/S41571-020-0356-4 32313224 PMC7901860

[B5] Cazzola M. Rogliani P. Aliberti S. Blasi F. Matera M. G. (2017). An Update On The Pharmacotherapeutic Management Of Lower Respiratory Tract Infections. Expert Opin. Pharmacother. 18, 973–988. doi: 10.1080/14656566.2017.1328497 28480770

[B6] De Pauw B. Walsh T. J. Donnelly J. P. Stevens D. A. Edwards J. E. Calandra T. (2008). Revised Definitions Of Invasive Fungal Disease From The European Organization For Research And Treatment Of Cancer/Invasive Fungal Infections Cooperative Group And The National Institute Of Allergy And Infectious Diseases Mycoses Study Group (Eortc/Msg) Consensus Group. Clin. Infect. Dis. 46, 1813–1821. doi: 10.1086/588660 18462102 PMC2671227

[B7] Duan H. Li X. Mei A. Li P. Liu Y. Li X.. (2021). The Diagnostic Value Of Metagenomic Next⁃Generation Sequencing In Infectious Diseases. BMC Infect. Dis. 21, 62. doi: 10.1186/S12879-020-05746-5 33435894 PMC7805029

[B8] Dumford D. Skalweit M. (2016). Antibiotic-Resistant Infections And Treatment Challenges In The Immunocompromised Host. Infect. Dis. Clin. North Am. 30, 465–489. doi: 10.1016/J.Idc.2016.02.008 27208768

[B9] Eichenberger E. M. De Vries C. R. Ruffin F. Sharma-Kuinkel B. Park L. Hong D.. (2021). Microbial Cell-Free Dna Identifies Etiology Of Bloodstream Infections, Persists Longer Than Conventional Blood Cultures, And Its Duration Of Detection Is Associated With Metastatic Infection In Patients With Staphylococcus Aureus And Gram-Negative Bacteremia. Clin. Infect. Dis. ciab742. doi: 10.1093/Cid/Ciab742 PMC9187311 34460909

[B10] Fan S. Ren H. Wei Y. Mao C. Ma Z. Zhang L.. (2018). Next-Generation Sequencing Of The Cerebrospinal Fluid In The Diagnosis Of Neurobrucellosis. Int. J. Infect. Dis. 67, 20–24. doi: 10.1016/J.Ijid.2017.11.028 29196276

[B11] Gasiorek M. Hsieh M. H. Forster C. S. (2019). Utility Of Dna Next-Generation Sequencing And Expanded Quantitative Urine Culture In Diagnosis And Management Of Chronic Or Persistent Lower Urinary Tract Symptoms. J. Clin. Microbiol. 58, e00204–19. doi: 10.1128/Jcm.00204-19 PMC6935904 31619534

[B12] Goldberg B. Sichtig H. Geyer C. Ledeboer N. Weinstock G. M. (2015). Making The Leap From Research Laboratory To Clinic: Challenges And Opportunities For Next-Generation Sequencing In Infectious Disease Diagnostics. Mbio 6, E01888–E01815. doi: 10.1128/Mbio.01888-15 26646014 PMC4669390

[B13] Grumaz C. Hoffmann A. Vainshtein Y. Kopp M. Grumaz S. Stevens P.. (2020). Rapid Next-Generation Sequencing-Based Diagnostics Of Bacteremia In Septic Patients. J. Mol. Diagn. 22, 405–418. doi: 10.1016/J.Jmoldx.2019.12.006 32146977

[B14] Grumaz S. Stevens P. Grumaz C. Decker S. O. Weigand M. A. Hofer S.. (2016). Next-Generation Sequencing Diagnostics Of Bacteremia In Septic Patients. Genome Med. 8, 73. doi: 10.1186/S13073-016-0326-8 27368373 PMC4930583

[B15] Gu W. Miller S. Chiu C. Y. (2019). Clinical Metagenomic Next-Generation Sequencing For Pathogen Detection. Annu. Rev. Pathol. 14, 319–338. doi: 10.1146/Annurev-Pathmechdis-012418-012751 30355154 PMC6345613

[B16] Haidar G. Singh N. (2022). Fever Of Unknown Origin. N. Engl. J. Med. 386, 463–477. doi: 10.1056/Nejmra2111003 35108471

[B17] Huang C. Chen H. Ding Y. Ma X. Zhu H. Zhang S.. (2021). A Microbial World: Could Metagenomic Next-Generation Sequencing Be Involved In Acute Respiratory Failure? Front. Cell Infect. Microbiol. 11. doi: 10.3389/Fcimb.2021.738074 PMC8522648 34671569

[B18] Huang J. Jiang E. Yang D. Wei J. Zhao M. Feng J.. (2020). Metagenomic Next-Generation Sequencing Versus Traditional Pathogen Detection In The Diagnosis Of Peripheral Pulmonary Infectious Lesions. Infect. Drug Resist. 13, 567–576. doi: 10.2147/Idr.S235182 32110067 PMC7036976

[B19] Íñigo M. Coello A. Fernández-Rivas G. Rivaya B. Hidalgo J. Quesada M. D.. (2016). Direct Identification Of Urinary Tract Pathogens From Urine Samples, Combining Urine Screening Methods And Matrix-Assisted Laser Desorption Ionization-Time Of Flight Mass Spectrometry. J. Clin. Microbiol. 54, 988–993. doi: 10.1128/Jcm.02832-15 26818668 PMC4809960

[B20] Kao R. L. Holtan S. G. (2019). Host And Graft Factors Impacting Infection Risk In Hematopoietic Cell Transplantation. Infect. Dis. Clin. North Am. 33, 311–329. doi: 10.1016/J.Idc.2019.02.001 30940461

[B21] Li Y. Sun B. Tang X. Liu Y. L. He H. Y. Li X. Y.. (2020). Application Of Metagenomic Next-Generation Sequencing For Bronchoalveolar Lavage Diagnostics In Critically Ill Patients. Eur. J. Clin. Microbiol. Infect. Dis. 39, 369–374. doi: 10.1007/S10096-019-03734-5 31813078 PMC7102353

[B22] Liu W. Fan Z. Zhang Y. Huang F. Xu N. Xuan L.. (2021). Metagenomic Next-Generation Sequencing For Identifying Pathogens In Central Nervous System Complications After Allogeneic Hematopoietic Stem Cell Transplantation. Bone Marrow Transplant. 56, 1978–1983. doi: 10.1038/S41409-021-01243-8 33824437 PMC8023769

[B23] Miao Q. Ma Y. Wang Q. Pan J. Zhang Y. Jin W.. (2018). Microbiological Diagnostic Performance Of Metagenomic Next-Generation Sequencing When Applied To Clinical Practice. Clin. Infect. Dis. 67, S231–S240. doi: 10.1093/Cid/Ciy693 30423048

[B24] Miller S. Naccache S. N. Samayoa E. Messacar K. Arevalo S. Federman S.. (2019). Laboratory Validation Of A Clinical Metagenomic Sequencing Assay For Pathogen Detection In Cerebrospinal Fluid. Genome Res. 29, 831–842. doi: 10.1101/Gr.238170.118 30992304 PMC6499319

[B25] Nie J. Yang L. Huang L. Gao L. Young K. H. Le Grange J. M.. (2022). Infection Complications In Febrile Chimeric Antigen Receptor (Car)-T Recipients During The Peri-Car-T Cell Treatment Period Examined Using Metagenomic Next-Generation Sequencing (Mngs). Cancer Commun. 10.1002/cac2.12260. doi: 10.1002/Cac2.12260 PMC9118034 35032364

[B26] Pendleton K. M. Erb-Downward J. R. Bao Y. Branton W. R. Falkowski N. R. Newton D. W.. (2017). Rapid Pathogen Identification In Bacterial Pneumonia Using Real-Time Metagenomics. Am. J. Respir. Crit. Care Med. 196, 1610–1612. doi: 10.1164/Rccm.201703-0537le 28475350 PMC5754443

[B27] Robilotti E. Holubar M. Seo S. K. Deresinski S. (2017). Feasibility And Applicability Of Antimicrobial Stewardship In Immunocompromised Patients. Curr. Opin. Infect. Dis. 30, 346–353. doi: 10.1097/Qco.0000000000000380 28542093

[B28] Sahin U. Toprak S. K. Atilla P. A. Atilla E. Demirer T. (2016). An Overview Of Infectious Complications After Allogeneic Hematopoietic Stem Cell Transplantation. J. Infect. Chemother. 22, 505–514. doi: 10.1016/J.Jiac.2016.05.006 27344206

[B29] Schlaberg R. Chiu C. Y. Miller S. Procop G. W. Weinstock G. (2017a). Validation Of Metagenomic Next-Generation Sequencing Tests For Universal Pathogen Detection. Arch. Pathol. Lab. Med. 141, 776–786. doi: 10.5858/Arpa.2016-0539-Ra 28169558

[B30] Schlaberg R. Queen K. Simmon K. Tardif K. Stockmann C. Flygare S.. (2017b). Viral Pathogen Detection By Metagenomics And Pan-Viral Group Polymerase Chain Reaction In Children With Pneumonia Lacking Identifiable Etiology. J. Infect. Dis. 215, 1407–1415. doi: 10.1093/Infdis/Jix148 28368491 PMC5565793

[B31] Schmidt-Hieber M. Silling G. Schalk E. Heinz W. Panse J. Penack O.. (2016). Cns Infections In Patients With Hematological Disorders (Including Allogeneic Stem-Cell Transplantation)-Guidelines Of The Infectious Diseases Working Party (Agiho) Of The German Society Of Hematology And Medical Oncology (Dgho). Ann. Oncol. 27, 1207–1225. doi: 10.1093/Annonc/Mdw155 27052648 PMC4922317

[B32] Schoch C. L. Ciufo S. Domrachev M. Hotton C. L. Kannan S. Khovanskaya R.. (2020). NCBI Taxonomy: A Comprehensive Update on Curation, Resources and Tools. Database 2020, baaa062. doi: 10.1093/database/baaa062 PMC7408187 32761142

[B33] Styczyński J. Tridello G. Koster L. Iacobelli S. Van Biezen A. Van Der Werf S.. (2020). Death After Hematopoietic Stem Cell Transplantation: Changes Over Calendar Year Time, Infections And Associated Factors. Bone Marrow Transplant. 55, 126–136. doi: 10.1038/S41409-019-0624-Z 31455899 PMC6957465

[B34] Tang W. Zhang Y. Luo C. Zhou L. Zhang Z. Tang X.. (2021). Clinical Application Of Metagenomic Next-Generation Sequencing For Suspected Infections In Patients With Primary Immunodeficiency Disease. Front. Immunol. 12. doi: 10.3389/Fimmu.2021.696403 PMC8414648 34484193

[B35] Tarabichi M. Shohat N. Goswami K. Parvizi J. (2018). Can Next Generation Sequencing Play A Role In Detecting Pathogens In Synovial Fluid? Bone Joint J. 100-B, 127–133. doi: 10.1302/0301-620x.100b2.Bjj-2017-0531.R2 29437053

[B36] Wang S. Ai J. Cui P. Zhu Y. Wu H. Zhang W. (2020). Diagnostic Value And Clinical Application Of Next-Generation Sequencing For Infections In Immunosuppressed Patients With Corticosteroid Therapy. Ann. Transl. Med. 8, 227. doi: 10.21037/Atm.2020.01.30 32309374 PMC7154484

[B37] Wang S. Chen Y. Wang D. Wu Y. Zhao D. Zhang J.. (2019). The Feasibility Of Metagenomic Next-Generation Sequencing To Identify Pathogens Causing Tuberculous Meningitis In Cerebrospinal Fluid. Front. Microbiol. 10. doi: 10.3389/Fmicb.2019.01993 PMC6733977 31551954

[B38] Watzinger F. Ebner K. Lion T. (2006). Detection And Monitoring Of Virus Infections By Real-Time Pcr. Mol. Aspects Med. 27, 254–298. doi: 10.1016/J.Mam.2005.12.001 16481036 PMC7112306

[B39] Wilson M. R. Sample H. A. Zorn K. C. Arevalo S. Yu G. Neuhaus J.. (2019). Clinical Metagenomic Sequencing For Diagnosis Of Meningitis And Encephalitis. N Engl. J. Med. 380, 2327–2340. doi: 10.1056/Nejmoa1803396 31189036 PMC6764751

[B40] Zhang H. C. Ai J. W. Cui P. Zhu Y. M. Hong-Long W. Li Y. J.. (2019). Incremental Value Of Metagenomic Next Generation Sequencing For The Diagnosis Of Suspected Focal Infection In Adults. J. Infect. 79, 419–425. doi: 10.1016/J.Jinf.2019.08.012 31442461

[B41] Zhang Y. Cui P. Zhang H. C. Wu H. L. Ye M. Z. Zhu Y. M.. (2020). Clinical Application And Evaluation Of Metagenomic Next-Generation Sequencing In Suspected Adult Central Nervous System Infection. J. Transl. Med. 18, 199. doi: 10.1186/S12967-020-02360-6 32404108 PMC7222471

