# Supplementary material for: Application of Next-Generation Sequencing in Infections After Allogeneic Haematopoietic Stem Cell Transplantation: A Retrospective Study
Source: Front Cell Infect Microbiol. 2022 Jun 14;12:888398. doi: 10.3389/fcimb.2022.888398 (PMC9239075; doi:10.3389/fcimb.2022.888398)
Supplement: Supplementary file 1 [file DataSheet_1.docx]

Supplementary Material

Supplementary methods

Protocol of conventional microbiological test

1. Bacterial and fungal smear and culture:

Gram stain, KOH test, and Ziehl-Neelsen stain were used to identify bacteria, fungi and Mycobacterium tuberculosis complex (MTBC) by smear microscopy. Samples were inoculated onto blood culture bottles. Simultaneously, the blood culture bottles were incubated in the BACTEC FX400 automated blood culture system until positivity or for a maximum of 5 days. Gram staining was done immediately after blood cultures signaled positive. The results of the Gram stains directed further subculturing onto relevant agar plates. These tests included catalase, oxidase, and indole spot tests and agglutination for Staphylococcus aureus, group A streptococci, and Streptococcus pneumoniae. The susceptibility testing was performed by disc diffusion according to the EUCAST method. Sabouraud dextrose agar supplemented with and without chloramphenicol were used to isolate fungi at 28 and 35◦C for up to 5 days, respectively. All cultured microorganisms were identified using the Bruker MALDI Biotyper (Bruker Corporation, Germany). Filamentous fungi were identified according to colony morphology and smear results;Enzyme-linked immune spot assay .

1. Enzyme-linked immune spot assayz

The T-SPOT kit was purchased from Oxford Immunotec Ltd. Samples of peripheral blood collected from all participants were analysed using the T-SPOT ELISpot assay according to the manufacturer’s instructions. Briefly, peripheral blood mononuclear cells (2.5- 3*10^5^) were added to 96-well plates precoated with anti-IFN-c antibody. Four wells were used for each patient: a positive control well to which PHA was added, a negative control well that contained the medium and two wells that contained TBAg ESAT-6 or CFP-10 peptide pools. Plates were incubated for 16–20 h at 37◦C with 5% carbon dioxide, washed with phosphate buffered saline and developed using an anti-IFN-c antibody conjugate and substrate to detect the presence of secreted IFN-c. Spot-forming cells (sfc) were counted with an automated ELISpot reader (CTL Analyzers, Cleveland, OH, USA). Positive and negative results were defined according to the manufacturer’s recommendations. Results were considered undetermined if the spot amounts in the positive control were 20 or 0.10 in the negative control in the negative control

3)Real-time PCR :

The RT-PCR were performed by available commercial kits. Total cell RNA was isolated, and 100 ng of each RNA sample was reverse transcribed and assayed by RT-PCR. Transcript quantification was performed with NMPA approved commercial kits. Duplicates were run for each sample in a 96-well plate. β-Actin was used as the endogenous reference gene. All quantitative RT-PCR reactions were run in three independent experiments. The relative quantification method was used, with the ratio of the mRNA level for the gene of interest normalized to the level of β-actin and the mean of control samples as the calibrator value. The specificity of the products was confirmed based on melting curves and electrophoresis.
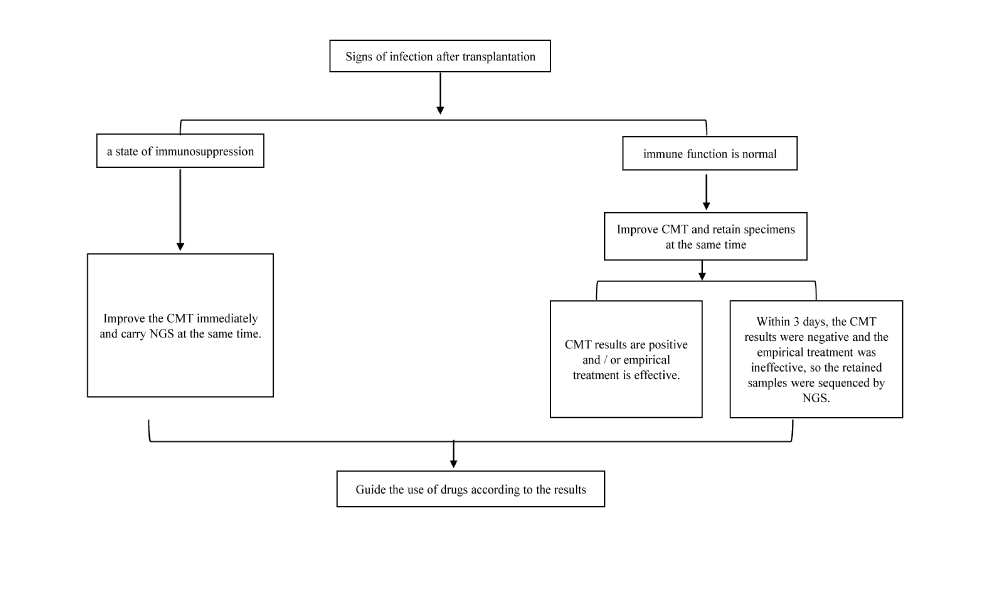


Supplementary Figure 1. Application principle of NGS in patients after HCST.

Supplementary Table 1. Patient’s characteristics of the three groups

| Patient characteristics | GROUP 1 | GROUP 2 | GROUP 3 |
| --- | --- | --- | --- |
| Num | 12 | 25 | 34 |
| Age, years (median, IQR) | 22(3.2-50） | 23(14-38) | 25.5(17-33） |
| Female | 9(75.0%) | 10(40.0%） | 18(51.9%) |
| Protopathy |  |  |  |
| AA | 2(16.7%) | 11(44.0%) | 8(23.5%) |
| ALL | 3(25.0%) | 5(20.0%) | 7(20.6%) |
| AML | 7(58.3%) | 7(28.0%) | 15(44.1%) |
| MDS | 0 | 0 | 3(8.8%) |
| T-LBL | 0 | 1(4.0%) | 1(4.0%) |
| Transplantation way |  |  |  |
| Haplo | 11(91.7%) | 23(92.0%) | 24(70.6%) |
| MSD | 0 | 0 | 6(17.6%) |
| MUD | 1(8.3%) | 2(8.0%) | 4(11.8%) |
| N engraftment,days (median，IQR) | 17(13-18) | 13(11-15) | 13(11-14) |
| PLT engraftment,days(median，IQR) | 13(11-18) | 12(11-13) | 14(12-15) |
| Main Symptoms |  |  |  |
| fever | 12(100%) | 21(84.0%） | 17(50.0%) |
| diarrhea | 0 | 1(4.0%） | 7(20.6%) |
| cough | 0 | 0 | 3(8.8%) |
| rash | 0 | 1(4.0%） | 2(5.9%) |
| blurred version | 0 | 0 | 1(2.9%) |
| headaches | 0 | 1(4.0%） | 2(5.9%) |
| ascites | 0 | 1(4.0%） | 0 |
| tic | 0 | 0 | 1(2.9%) |
| Agranulocytic | 12(100%) | 6(24.0%) | 5(14.7%) |
| Immunosuppressive drugs | 12(100%) | 22(88.0%) | 24(70.6%) |
| GVHD with coinfection | 0 | 7 | 9 |
| NGS pre-positive | 6（50%） | 17(68%) | 17(50.0%) |

Abbreviations: GVHD, graft versus host disease.
